# Comparative gender peptidomics of *Bothrops atrox* venoms: are there differences between them?

**DOI:** 10.1590/1678-9199-JVATITD-2020-0055

**Published:** 2020-10-07

**Authors:** Adriana Simizo, Eduardo S. Kitano, Sávio S. Sant’Anna, Kathleen Fernandes Grego, Anita Mitico Tanaka-Azevedo, Alexandre K. Tashima

**Affiliations:** 1 Department of Biochemistry, Federal University of São Paulo (Unifesp), São Paulo, SP, Brazil.; 2 Laboratory of Immunology, Heart Institute, Medical School, University of São Paulo (USP), São Paulo, SP, Brazil.; 3 Laboratory of Herpetology, Butantan Institute, São Paulo, SP, Brazil.; 4 Special Laboratory for Applied Toxinology, Center of Toxins, Immune-Response and Cell Signaling, Butantan Institute, São Paulo, SP, Brazil.

**Keywords:** Bothrops atrox, Venom, Peptidomics, Sexual dimorphism, Disintegrin

## Abstract

**Background:**

*Bothrops atrox* is known to be the pit viper responsible for most snakebites and human fatalities in the Amazon region. It can be found in a wide geographical area including northern South America, the east of Andes and the Amazon basin. Possibly, due to its wide distribution and generalist feeding, intraspecific venom variation was reported by previous proteomics studies. Sex-based and ontogenetic variations on venom compositions of *Bothrops* snakes were also subject of proteomic and peptidomic analysis. However, the venom peptidome of *B. atrox* remains unknown.

**Methods:**

We conducted a mass spectrometry-based analysis of the venom peptides of individual male and female specimens combining bottom-up and top-down approaches.

**Results:**

We identified in *B. atrox* a total of 105 native peptides in the mass range of 0.4 to 13.9 kDa. Quantitative analysis showed that phospholipase A_2_ and bradykinin potentiating peptides were the most abundant peptide families in both genders, whereas disintegrin levels were significantly increased in the venoms of females. Known peptides processed at non-canonical sites and new peptides as the Ba1a, which contains the SVMP BATXSVMPII1 catalytic site, were also revealed in this work.

**Conclusion:**

The venom peptidomes of male and female specimens of *B. atrox* were analyzed by mass spectrometry-based approaches in this work. The study points to differences in disintegrin levels in the venoms of females that may result in distinct pathophysiology of envenomation. Further research is required to explore the potential biological implications of this finding.

## Background

Snake venoms are toxic glandular secretions containing high concentrations of proteins and peptides. Their biologically active components were elaborated and refined over millions of years of evolution through an arms race with its preys [1,2]. Particularly, *Bothrops atrox* is a highly adapted and widely distributed species found in many countries of northern South America [3,4]. The snake is responsible for most snakebites in Northern Brazil [5] and its venom is characterized by three main pathophysiological activities: coagulant, hemorrhagic, and acute inflammatory effects [6]. Previous proteomics studies revealed intraspecific variation in *B. atrox* venom composition associated to its wide geographical distribution range [3,4,7]. Differences and similarities in venom compositions were found in these studies, suggesting that venom phenotypes may be classified according to specific regions [4]. Other intraspecific venom variations related to sex [8-10], diet [11,12] and ontogeny [13] are well-documented phenomena in snake species. A remarkable sexual dimorphism in *B. atrox* is the size difference between males and females [14]. Males are significantly smaller than females and as a result may present higher motilities [14]. 

On the other side of venom research, toxins have been sources of inspiration for drug research and also significant in elucidating major biochemical and physiological mechanisms in vertebrates [15-17]. Biochemical approaches of isolation and analysis of purified venom components revealed important biologically active peptides including the bradykinin potentiating peptides (BPPs) [18,19], sarafotoxins [20], disintegrins [21] and analgesic peptides as crotalphine [22], for instance. However, despite the discovery of important venom toxins and the maturity achieved by the snake venomics [23] and other venom proteomics approaches [24,25], venom peptidomics is still an emerging research field [17,26]. Only a few peptides have been characterized in the venom of *B. atrox*, as the BPP-12a, BPP-BAX12 [27,28] and the disintegrin batroxostatin [21]. 

Peptidomics analysis of other *Bothrops* snake venoms revealed new BPPs, poly-His-poly-Gly peptides and other protein fragments [13,29,30]. Biological assays indicated that few amino acid mutations have significant effects on the activities of peptides within the same class [29]. Thus, considering the richness of the yet unexplored peptidome and the sexual dimorphism of *B. atrox*, we used in this work a combination of mass spectrometry-based analysis and bioinformatics to compare the male and female *Bothrops atrox* venom peptidomes.

## Methods

### Reagents

Proteolytic enzymes (Asp-N, Glu-C and trypsin) were purchased from Promega. Dithiothreitol (DTT) and iodoacetamide were obtained from GE Healthcare. Acetonitrile was purchased from Avantor Pierce. Unless otherwise stated, all other reagents were acquired from Sigma-Aldrich.

### Animals

Adult *Bothrops atrox* specimens from Northeastern of Brazil (Viana, MA) were maintained in the biotherium of the Laboratório de Herpetologia, Instituto Butantan (SP, Brazil). Experiments were approved by the Ethical Committee of Instituto Butantan (number 6303280220), the Ethical Committee of Universidade Federal de São Paulo (number 3437250719) and performed in accordance to the Brazilian laws for the use of experimental animals and with the ethical principles adopted by the Brazilian College of Animal Experimentation (COBEA). 

### Venom extraction and fractionation

Venom samples were extracted from four female and four male specimens of *B. atrox*. The animals were previously anesthetized with carbon dioxide. Venoms were individually extracted into beakers kept in ice bath and immediately mixed with the proteinase inhibitors EDTA and PMSF to final concentrations of 5 mM and 2 mM, respectively [29,31]. The venom solutions were centrifuged at 16,000 g and 4 °C for 5 min to remove debris, lyophilized and stored at -20 °C for further fractionation.

Venom peptidomic fractions were obtained from 50 µg aliquots of crude venoms subjected to solid-phase extraction with C18 stage tips and eluted with 40% ACN [32]. Stage tips were assembled with InertSep RP-C18 resin (GL Sciences) and SDB-XC membrane (Empore, 3M) inside P200 pipette tips. The eluates were dried in a vacuum concentrator (Concentrator Plus, Eppendorf) and the dried venom eluates were stored at -20 °C until MS analysis or digestion prior to analysis. 

### Enzyme digestion

Pools of venoms (males and females distinctly) containing 50 µg of crude venom each were separately digested with three different enzymes. Venoms were dissolved in specific buffer solutions for each enzyme and digested as previously described [33,34]. Briefly, for trypsin and Asp-N, samples were dissolved in 50 mM NH_4_HCO_3_ and in 50 mM sodium phosphate for digestion with Glu-C. Additionally, two other pools of each gender containing 100 µg of crude venom were digested only with trypsin. The enzyme to protein ratio of 1:100 was used for all digestions. Samples were incubated with 0.2% RapiGest surfactant (Waters) at 80 °C for 15 min, followed by centrifugation at 2000 g for 3 min. All samples were reduced with 5 mM DTT for 30 min at 60 °C and alkylated with 10 mM iodoacetamide for 30 min in the dark at room temperature. Incubations with the enzymes were conducted for 30 minutes at 37 °C. TFA at final concentration of 0.5% was added to the samples to stop the digestions and to cleave the RapiGest surfactant. Samples were cleaned in stage tips, as described in the fractionation section, before LC-MS/MS analysis.

### Mass spectrometry acquisition

LC-MS/MS analysis of native and digested toxins were performed on a Synapt G2 HDMS mass spectrometer (Waters) coupled to a nanoAcquity UPLC (Waters) chromatographic system. Samples were injected into a trap column (nanoAquity C18 trap column Symmetry 180 µm x 20 mm, Waters) and transferred by an elution gradient to an analytical column (nanoAcquity C18 BEH 75 µm x 150 mm, 1.7 mm, Waters). Mobile phase A (0.1% formic acid in water) and B (0.1% formic acid in acetonitrile) were used to generate a 7-35% B elution gradient run over 60 min at a flow rate of 275 nl/min. Data were acquired in the data-independent acquisition modes MS^E^ and UDMS^E^ with ion mobility separation [33,35], in the m/z range of 50-2000 and operating in resolution mode. Peptide ions were fragmented by collision induced dissociation (CID) switching from low (4 eV) to high (ramped from 19 to 45 eV) collision energy, for accurate measurement of both precursor and fragment ions. Scan times were set to 1.25 s. The ESI source was operated in the positive mode with a capillary voltage of 3.0 kV, block temperature of 100 °C and cone voltage of 40 V. Glu-fibrinopeptide B (Peptide 2.0) was infused through the nanoLockSpray source and sampled for 1 s every 60 s for external calibration. Native venom peptides and digested samples were analyzed in technical duplicates, totalizing 46 LC-MS/MS runs. 

### Bioinformatics analysis

*Quantitative analysis of native peptides*

Raw data of native peptides were processed and analyzed in Progenesis QI for Proteomics (Nonlinear Dynamics). Relative quantification and retention time alignment were based on peptide ion data of a reference run automatically selected. Only native peptide ions with normalized abundance above 200 counts and detected in at least 2 biological replicates of male or female groups were considered for further analysis. Entries with differences in monoisotopic mass and retention time below 30 ppm and 2 min, respectively, were regarded as redundant and only the entry with higher average abundance was considered.

*Peptide identification*

MS/MS spectra peak lists were generated in the software ProteinLynx Global Server 3.0.3 (Waters) as .mzML files. Spectra were processed by the Apex3D module using low energy threshold of 750 counts and high energy threshold of 50 counts. The peak lists of native peptide samples were submitted to searches using MASCOT 2.2.04 (Matrix Science) and PEAKS Studio 7.5 (Bioinformatics Solution Inc.) against the following Uniprot databases: *Bothrops atrox* with 202 entries (date of fasta file: June 21, 2018), *Bothrops* with 1,120 entries (date of fasta file: June 21, 2018) and Serpentes with 156,483 entries (date of fasta file: May 28, 2020). The search parameters set in PEAKS Studio were: no enzyme specificity, pyroglutamic acid from N-terminal Gln or Glu and methionine oxidation as variable modifications, mass tolerances of 10 ppm for precursor ions and 0.025 Da for fragments ions and FDR of 1% at the peptide level. *De novo* (ALC ≥ 50%), post-translational modifications (PEAKS PTM) and homology (SPIDER module) searches were also performed in PEAKS Studio. The same database and variable modifications were set on MASCOT engine. Peptide and fragment mass tolerances were set to 0.1 Da and ion identifications were considered for expectation values lower than 0.05 (p < 0.05). The expectation cut-off value of 0.05 was applied in the MASCOT ion score to avoid peptide identifications out of the 95% confidence interval to be selected.

The MS/MS spectra of digested samples were submitted to database search in PEAKS Studio using the same databases and mass tolerances. Enzyme specificity was defined for each sample and up to one non-specific and three missed cleavages were allowed per peptide. Carbamidomethylation of Cys was set as fixed modification and Met oxidation, N-terminus acetylation and Asn/Gln deamidation were set as variable modifications. 

*Native peptidome analysis*

Identified peptides from digested samples on PEAKS Studio were manually reviewed and N- and C-terminii from native peptides were determined by consensus of non-specific cleavages and overlapping peptides. To validate the native sequences of heavy peptides (> 5 kDa), identified by overlapping cleaved peptides in PEAKS Studio, the experimental mass of each ion was compared to its theoretical mass calculated in ProteinProspector v 5.22.1 (http://prospector.ucsf.edu/prospector/mshome.htm). For peptides < 8 kDa, the monoisotopic masses were used in the comparisons. The sequences were validated if the relative mass difference in ppm was equal or less than 30 and a minimum of 4 fragments matching b+ or y+ ion series were found [36,37]. For peptides ( 8 kDa, the average masses were used in the comparisons and the sequences were validated if the relative mass difference in ppm was equal or less than 200 ppm.

*Peptide alignment*

Primary structures of selected peptides were analyzed by homology searches using protein BLAST (https://www.uniprot.org/blast/) and aligned in TCoffee [38] or in PEAKS Studio 7.5.

### Peptide folding and visualization

The three-dimensional structure of peptide sequences were predicted by PEP-FOLD3 [39] using default parameters. Structure visualization and comparison with other proteins were performed in PyMOL Molecular Graphics System, Version 2.3.4 (Schrödinger, LLC).

## Results 

### **Identification of *B. atrox* venom peptides**

**Figure 1.**
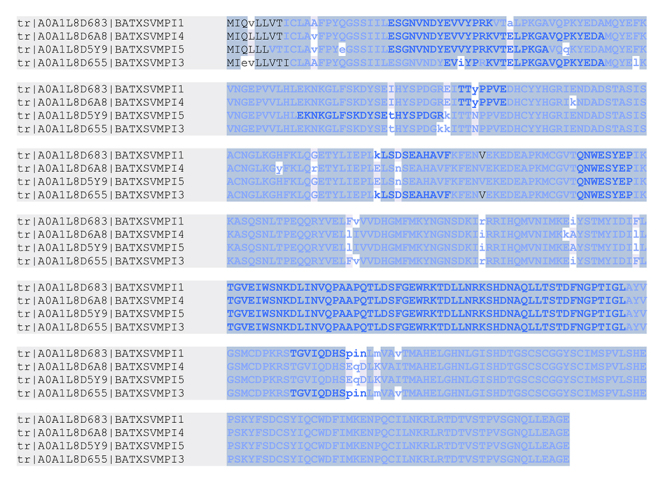
Alignment of the SVMPI peptides identified in the venoms of *B. atrox* with the precursor proteins BATXSVMPI1, BATXSVMPI4, BATXSVMPI5 and BATXSVMPI3. The peptides are shared among several of the homologous proteins. Letters in bold blue represent the amino acids covered by MS/MS spectra. Capital letters represent complete identity among the aligned sequences. Alignment performed in PEAKS Studio 7.5.

The biometric data of the specimens used for venom extraction are shown in Table 1. Native and digested *B. atrox* venom peptidome samples were analyzed by LC-MS/MS in the data-independent acquisition mode. The native samples of female and male groups were analyzed individually, totalizing 16 runs (4 individual samples for each group and analyzed in technical duplicates). Processing of the raw data in Progenesis QI for Proteomics resulted in 4112 features detected, that after application of the inclusion criteria were reduced to 878 precursor ions (Additional file 1). Automated *de novo* analysis, followed by database search resulted in 375 peptide-spectrum matches (PSM) from 88 unique peptides and 31 precursor proteins (Table 2 and Additional file 2). Three additional peptides were sequenced by *de novo* analysis, summing 91 (Table 2). Most of the peptides were from the SVMP family, 46 from SVMPI, 24 from SVMPII and 7 from SVMPIII. The other 14 were BPPs (Table 2). The metalloprotease BATXSVMPI1 contributed with the majority of the SVMPI peptides, covering 39 of the identified peptides, followed by the BATXSVMPI3, BATXSVMPI4 and BATXSVMPI5 with 37 peptides (Additional file 2). Several of the peptides are shared among these homologous toxins (Figure 1 and Additional file 2). The same is true for the SVMPII and SVMPIII peptides (Additional file 2). 

**Table 1.** Biometric data of the *B. atrox* specimens used for venom extraction. Size 1 is the length from the head to the cloaca and Size 2 is the total length of the animal.

Snake	Sex	Weight (g)	Size 1 (cm)	Size 2 (cm)
F1	♀	250	78	89
F2	♀	365	91	104
F3	♀	585	94	108
F4	♀	270	83	94
M1	♂	260	92	107
M2	♂	275	87	102
M3	♂	215	74	86
M4	♂	250	81	94

**Table 2.** Native peptides identified in the venoms of female and male specimens of *B. atrox* by LC-MS/MS analysis, bottom-up and top-down fragmentation, *de novo* sequencing, intact mass deconvolution and database search.

m/z	RT (min)	Mass (Da)	z	ID^a^	Protein accession	Peptide sequence^b^	Description	Protein family	FC^c^	t-test
547.62	17.12	1,639.8	3	P	A0A1L8D662	DLRPDGKQARQNWG	BATXBPP10	BPP	4.04	0.2025
609.34	32.05	608.3	1	P	A0A1L8D662	PGPEIP	BATXBPP10	BPP	2.29	0.0433
706.38	23.29	705.4	1	P	A0A1L8D662	PGPEIPP	BATXBPP10	BPP	0.62	0.6662
445.17	30.04	444.2	1	Dn	A0A1L8D662	ZKW	BATXBPP10	BPP	0.96	0.8204
612.32	23.93	611.3	1	P	A0A1L8D662	ZKWAP	BATXBPP10	BPP	3.00	0.3162
541.28	24.68	540.3	1	Dn	A0A1L8D662	ZKWP	BATXBPP10	BPP	1.41	0.3686
644.34	34.85	1,286.7	2	P	A0A1L8D662	ZKWPRPGPEIP	BATXBPP10	BPP	0.43	0.0969
692.88	33.34	1,383.7	2	P	A0A1L8D662	ZKWPRPGPEIPP	BATXBPP10	BPP	0.55	0.3568
799.94	43.14	1,597.9	2	P	A0A1L8D662	ZKWPRPGPEIPPLT	BATXBPP10	BPP	13.83	0.0531
525.30	31.17	1,048.6	2	P	A0A1L8D680	ZKWPSPKVP	BATXBPP11	BPP	0.96	0.4366
573.82	31.04	1,145.6	2	P	A0A1L8D680	ZKWPSPKVPP	BATXBPP11	BPP	1.58	0.4048
949.48	40.69	1,897.0	2	P	A0A1L8D662	ZQWAQKWPRPGPEIPP	BATXBPP10	BPP	20.82	0.1444
978.47	48.10	977.5	1	P	A0A1L8D662	ZSWPGPNIP	BATXBPP10	BPP	1.30	0.4257
1075.52	45.40	1,074.5	1	P	A0A1L8D5X1	ZSWPGPNIPP	BATXBPP10	BPP	1.05	0.6470
929.26	20.03	7,426.0	8	Bu/Td	A0A1L8D600	AGEECDCGAPENPCCDAATCKLRPG...	BATXDIS1:2-71	DIS	14.95	1.64E-06
955.28	19.25	7,634.2	8	Bu/Td	A0A1L8D600	AGEECDCGAPENPCCDAATCKLRPG...	BATXDIS1:2-73	DIS	1.05	0.4170
943.14	18.25	7,537.0	8	Bu/Td	A0A1L8D600	ZAGEECDCGAPENPCCDAATCKLRP...	BATXDIS1:Z1-71	DIS	10.56	6.91E-10
1022.59	18.58	7,151.0	7	Bu/Td	A0A1L8D600	ZCDCGAPENPCCDAATCKLRPGAQC...	BATXDIS1:Z5-71	DIS	1.08	0.3716
809.90	21.59	7,280.0	9	Bu/Td	A0A1L8D600	ZECDCGAPENPCCDAATCKLRPGAQ...	BATXDIS1:Z4-71	DIS	8.02	0.0250
1080.32	18.25	7,555.2	7	Bu/Td	A0A1L8D600	EAGEECDCGAPENPCCDAATCKLRP...	BATXDIS1:1-71	DIS	7.05	3.85E-07
1110.04	19.50	7,763.2	7	Bu/Td	A0A1L8D600	EAGEECDCGAPENPCCDAATCKLRP...	BATXDIS1	DIS	0.64	0.7065
897.13	19.54	7,169.0	8	Bu/Td	A0A1L8D600	ECDCGAPENPCCDAATCKLRPGAQC...	BATXDIS1:5-71	DIS	8.71	0.0354
1054.88	18.88	7,377.1	7	Bu/Td	A0A1L8D600	ECDCGAPENPCCDAATCKLRPGAQC...	BATXDIS1:5-73	DIS	0.30	0.8193
913.27	19.84	7,298.1	8	Bu/Td	A0A1L8D600	EECDCGAPENPCCDAATCKLRPGAQ...	BATXDIS1:4-71	DIS	4.74	1.79E-07
1073.31	19.20	7,506.1	7	Bu/Td	A0A1L8D600	EECDCGAPENPCCDAATCKLRPGAQ...	BATXDIS1:4-73	DIS	0.30	0.4403
920.38	19.84	7,355.0	8	Bu/Td	A0A1L8D600	GEECDCGAPENPCCDAATCKLRPGA...	BATXDIS1:3-71	DIS	9.36	5.88E-06
1379.25	58.88	13,782.5	10	Bu/Td/Dn	BATROXPLA2X	HLVQFEKLLQLLAGR*	PLA2	PLA2	0.61	0.2741
1262.26	52.23	13,873.8	11	Bu/Td/Dn	A0A1L8D605	SLIEFANMILEETKKSPFPYYTTYG...	BATXPLA6	PLA2	0.59	0.3789
493.24	32.56	1,476.7	3	P	A0A1L8D683	AAPQTLDSFGEWR	BATXSVMPI1	SVMPI	2.57	0.0280
580.31	38.69	2,317.2	4	P	A0A1L8D683	AAPQTLDSFGEWRKTDLLNR	BATXSVMPI1	SVMPI	0.02	0.1218
448.70	25.80	895.4	2	P	A0A1L8D683	DSFGEWR	BATXSVMPI1	SVMPI	2.42	0.0089
579.62	32.09	1,735.8	3	P	A0A1L8D683	DSFGEWRKTDLLNR	BATXSVMPI1	SVMPI	0.04	0.2121
598.06	35.52	2,388.2	4	P	A0A1L8D683	DSFGEWRKTDLLNRKSHDNA	BATXSVMPI1	SVMPI	0.81	0.8959
630.07	25.29	2,516.2	4	P	A0A1L8D683	DSFGEWRKTDLLNRKSHDNAQ	BATXSVMPI1	SVMPI	0.70	0.9531
897.98	41.73	1,794.0	2	P	A0A1L8D683	EIWSNKDLINVQPAAP	BATXSVMPI4	SVMPI	1.03	0.5521
492.44	18.84	2,457.2	5	P	A0A1L8D5Y9	EKNKGLFSKDYSETHYSPDGR	BATXSVMPI5	SVMPI	13.29	0.1150
742.41	25.38	741.4	1	P	A0A1L8D5Y8	ERDLLP	BATXSVMPI6	SVMPI	1.14	0.2547
606.32	22.16	605.3	1	P	A0A1L8D683	EVVYP	BATXSVMPI1	SVMPI	1.11	0.6172
512.28	27.85	1,533.8	3	P	A0A1L8D683	FGEWRKTDLLNR	BATXSVMPI1	SVMPI	0.04	0.1370
547.53	22.80	2,186.1	4	P	A0A1L8D683	FGEWRKTDLLNRKSHDNA	BATXSVMPI1	SVMPI	0.73	0.8092
647.35	36.13	1,292.7	2	P	A0A1L8D683	FLTGVEIWSNK	BATXSVMPI1	SVMPI	0.72	0.3578
463.25	29.13	1,386.7	3	P	A0A1L8D683	GEWRKTDLLNR	BATXSVMPI1	SVMPI	0.25	0.0871
1268.59	34.90	1,267.6	1	P	A0A1L8D683	GNVNDYEVVYP	BATXSVMPI1	SVMPI	7.86	0.0606
540.28	21.59	1,078.5	2	P	A0A1L8D683	GVIQDHSPIN	BATXSVMPI1	SVMPI	0.18	0.0552
596.82	35.60	1,191.6	2	M	A0A1L8D683	GVIQDHSPINL	BATXSVMPI1	SVMPI	1.03	0.5952
542.47	18.63	2,707.3	5	M	A0A1L8D5Y9	HLEKNKGLFSKDYSETHYSPDGR	BATXSVMPI5	SVMPI	6.49	0.1359
602.31	22.59	1,202.6	2	P	A0A1L8D683	KLSDSEAHAVF	BATXSVMPI1	SVMPI	4.43	0.1596
706.02	35.30	2,115.0	3	P	A0A1L8D683	KSHDNAQLLTSTDFNGPTIG	BATXSVMPI1	SVMPI	0.01	0.1035
743.71	44.23	2,228.1	3	P	A0A1L8D683	KSHDNAQLLTSTDFNGPTIGL	BATXSVMPI1	SVMPI	0.03	0.0656
625.01	20.33	1,872.0	3	P	A0A1L8D6A8	KVTELPKGAVQPKYEDA	BATXSVMPI4	SVMPI	0.95	0.4174
626.32	35.47	2,501.3	4	P	A0A1L8D683	LDSFGEWRKTDLLNRKSHDNA	BATXSVMPI1	SVMPI	0.66	0.9175
658.33	35.26	2,629.3	4	P	A0A1L8D683	LDSFGEWRKTDLLNRKSHDNAQ	BATXSVMPI1	SVMPI	0.87	0.6366
689.86	41.05	2,755.4	4	P	A0A1L8D683	NVQPAAPQTLDSFGEWRKTDLLNR	BATXSVMPI1	SVMPI	0.03	0.1134
1221.54	44.35	1,220.5	1	P	A0A1L8D683	ZTLDSFGEWR	BATXSVMPI1	SVMPI	3.94	0.0035
783.36	37.18	1,564.7	2	P	A0A1L8D683	ZTLDSFGEWRKTD	BATXSVMPI1	SVMPI	0.49	0.5500
839.92	43.99	1,677.8	2	P	A0A1L8D683	ZTLDSFGEWRKTDL	BATXSVMPI1	SVMPI	0.66	0.8517
953.47	47.53	1,904.9	2	P	A0A1L8D683	ZTLDSFGEWRKTDLLN	BATXSVMPI1	SVMPI	0.02	0.1090
688.02	42.78	2,061.0	3	P	A0A1L8D683	ZTLDSFGEWRKTDLLNR	BATXSVMPI1	SVMPI	0.05	0.0986
679.34	35.47	2,713.3	4	P	A0A1L8D683	ZTLDSFGEWRKTDLLNRKSHDNA	BATXSVMPI1	SVMPI	0.95	0.8715
711.35	35.26	2,841.4	4	P	A0A1L8D683	ZTLDSFGEWRKTDLLNRKSHDNAQ	BATXSVMPI1	SVMPI	1.18	0.5545
625.26	33.73	624.3	1	P	A0A1L8D683	SFGEW	BATXSVMPI1	SVMPI	4.52	0.0392
391.19	22.89	780.4	2	P	A0A1L8D683	SFGEWR	BATXSVMPI1	SVMPI	3.45	0.0305
541.28	29.09	1,620.8	3	P	A0A1L8D683	SFGEWRKTDLLNR	BATXSVMPI1	SVMPI	0.12	0.1099
601.31	22.50	2,401.2	4	P	A0A1L8D683	SFGEWRKTDLLNRKSHDNAQ	BATXSVMPI1	SVMPI	0.62	0.7213
756.36	27.30	1,510.7	2	P	A0A1L8D683	SGNVNDYEVVYPR	BATXSVMPI1	SVMPI	3.30	0.5639
547.27	22.46	1,638.8	3	P	A0A1L8D683	SGNVNDYEVVYPRK	BATXSVMPI1	SVMPI	3.44	0.0348
1026.53	46.10	2,051.1	2	P	A0A1L8D683	TGVEIWSNKDLINVQPAAP	BATXSVMPI1	SVMPI	0.73	0.3924
1141.10	44.31	2,280.2	2	P	A0A1L8D683	TGVEIWSNKDLINVQPAAPQT	BATXSVMPI1	SVMPI	1.40	0.5050
590.81	22.25	1,179.6	2	P	A0A1L8D683	TGVIQDHSPIN	BATXSVMPI1	SVMPI	0.16	0.1503
1109.51	28.68	1,108.5	1	P	A0A1L8D683	TSTDFNGPTIG	BATXSVMPI1	SVMPI	0.34	0.0927
1222.59	41.82	1,221.6	1	P	A0A1L8D683	TSTDFNGPTIGL	BATXSVMPI1	SVMPI	0.68	0.3285
947.51	44.27	1,893.0	2	P	A0A1L8D683	VEIWSNKDLINVQPAAP	BATXSVMPI1	SVMPI	0.29	0.0164
401.23	18.84	1,200.7	3	P	A0A1L8D683	WRKTDLLNR	BATXSVMPI1	SVMPI	0.05	0.2819
604.00	17.41	2,412.0	4	M	A0A1L8D5Y8	WVHEMVNSMNGFYRPMDMH	BATXSVMPI6	SVMPI	11.02	0.0606
555.58	18.58	1,663.7	3	P	A0A1L8D600	AHELGHNLGMYHDGN	Ba1a (BATXSVMPII1)	SVMPII	5.76	0.0629
613.37	22.42	612.4	1	Dn	A0A1L8D600	DLLPR	BATXSVMPII1	SVMPII	1.38	0.3668
475.78	29.47	949.5	2	P	A0A1L8D600	DLLPRISH	BATXSVMPII1	SVMPII	5.94	0.1492
499.24	32.56	1,494.7	3	P	A0A1L8D600	DS(-18.01)SKTLTSFGEWR	BATXSVMPII1	SVMPII	4.40	0.0264
757.38	32.56	1,512.7	2	P	A0A1L8D600	DSSKTLTSFGEWR	BATXSVMPII1	SVMPII	7.02	0.0048
600.30	28.51	1,797.9	3	P	A0A1L8D600	DSSKTLTSFGEWRER	BATXSVMPII1	SVMPII	1.33	0.6645
528.65	22.42	1,582.9	3	P	A0A1L8D600	ZVWSKKDLIKVEK	BATXSVMPII1	SVMPII	4.38	0.0598
616.53	38.43	3,077.6	5	P	A0A1L8D600	ZVWSKKDLIKVEKDSSKTLTSFGEW	BATXSVMPII	SVMPII	-	0.0211
610.31	24.38	609.3	1	M	A0A1L8D600	EVLSY	BATXSVMPII1	SVMPII	0.44	0.8581
502.77	19.84	1,003.5	2	P	A0A1L8D600	EVWSKKDL	BATXSVMPII1	SVMPII	2.69	0.0083
534.65	22.42	1,600.9	3	P	A0A1L8D600	EVWSKKDLIKVEK	BATXSVMPII1	SVMPII	8.83	0.0583
620.13	38.43	3,095.6	5	P	A0A1L8D600	EVWSKKDLIKVEKDSSKTLTSFGEW	BATXSVMPII1	SVMPII	12.74	0.0110
619.83	23.38	1,237.7	2	P	A0A1L8D600	GVVRDHSEINL	BATXSVMPII1	SVMPII	9.27	0.0122
683.86	20.12	1,365.7	2	P	A0A1L8D600	GVVRDHSEINLQ	BATXSVMPII1	SVMPII	9.03	0.0037
528.53	28.59	2,110.1	4	P	A0A1L8D600	IKVEKDSSKTLTSFGEWR	BATXSVMPII1	SVMPII	3.60	0.0135
599.82	25.93	2,395.3	4	P	A0A1L8D600	IKVEKDSSKTLTSFGEWRER	BATXSVMPII1	SVMPII	1.70	0.3892
500.26	25.59	1,997.0	4	P	A0A1L8D600	KVEKDSSKTLTSFGEWR	BATXSVMPII1	SVMPII	-	0.0575
571.55	22.72	2,282.2	4	P	A0A1L8D600	KVEKDSSKTLTSFGEWRER	BATXSVMPII1	SVMPII	1.20	0.4292
556.80	31.98	2,223.2	4	P	A0A1L8D600	LIKVEKDSSKTLTSFGEWR	BATXSVMPII1	SVMPII	1.47	0.2568
576.99	37.35	1,727.9	3	M	A0A1L8D649	LQGETYLIEPLKLPD	BATXSVMPII7	SVMPII	11.24	0.0447
548.23	15.58	1,641.7	3	P	A0A1L8D5Z6	SEDYSETHYSPDGR	BATXSVMPII3	SVMPII	1.84	0.0759
656.34	29.51	1,310.7	2	P	A0A1L8D600	SKTLTSFGEWR	BATXSVMPII1	SVMPII	11.48	0.0753
532.95	24.93	1,595.8	3	P	A0A1L8D600	SKTLTSFGEWRER	BATXSVMPII1	SVMPII	1.48	0.5138
441.71	24.68	881.4	2	P	A0A1L8D600	TSFGEWR	BATXSVMPII1	SVMPII	23.84	0.0084
458.61	22.38	1,372.8	3	M	A0A1L8D5W6	KLNKPTIGIAYR	BATXSVMPIII21	SVMPIII	7.75	0.0468
1135.51	31.81	1,134.5	1	P	A0A1L8D5Z1	ZTNWKSYEP	BATXSVMPIII3	SVMPIII	1.24	0.4209
398.19	23.72	794.4	2	P	A0A0K2JNB8	SFAEWR	BATROXRHAGIN	SVMPIII	0.38	0.7499
626.82	28.47	1,251.6	2	P	A0A0K2JNB8	SFAEWRKTDL	BATROXRHAGIN	SVMPIII	0.51	0.3288
541.62	30.60	1,621.8	3	P	A0A0K2JNB8	SFAEWRKTDLLTR	BATROXRHAGIN	SVMPIII	0.03	0.1568
634.81	23.38	1,267.6	2	P	A0A0K2JNB8	TAIDFNGPTIGY	BATROXRHAGIN	SVMPIII	1.42	0.1891
632.82	25.55	1,263.6	2	P	A0A0K2JNB8	TVKPDVDYTLN	BATROXRHAGIN	SVMPIII	0.31	0.2642

^a^ Obtained from: P - PEAKS Studio; M - Mascot Server; Dn - *de novo* analysis; Bu - bottom-up; Td - top-down. ^b^Only the first 25 amino acids are shown. Complete sequences in Additional file 1. ^c^Fold change.

Additional searches with the *Bothrops* and Serpentes databases resulted in 119 and 63 unique peptides, respectively (Venn diagram [40] in Additional file 3). The *Bothrops* database resulted in more peptide identifications. However, opposed to the expected, the Serpentes database (that contains all *Bothrops* sequences) resulted in less identifications. The decrease can be explained by the exponential expansion of the search space for peptidomics searches [41], as the sensitivity of a peptide-spectrum match search tool varies inversely with the size of the sequence database [42]. As a result, the number of identifications decreases. Furthermore, when we applied the inclusion criteria of ion intensity and presence in biological replicates, the list of relevant peptides of the *B. atrox* and *Bothrops* databases did not differ.

**Figure 2.**
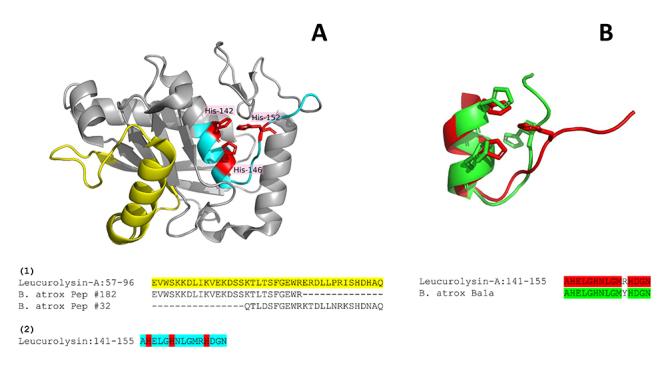
(A) Ribbon diagram of the SVMPI leucurolysin-A 3D structure (4Q1L [44]). The region in yellow (1) corresponds to the native homologous peptides Pep #182 and Pep #32 (Additional file 1) identified in *Bothrops atrox* venoms. The sequence highlighted in cyan (2) corresponds to the homologous peptide Ba1a, which aligns to the catalytic site containing the three Zn-binding histidines (red). (B) Ba1a fold predicted by PEP-FOLD3 (in green) compared to the original crystallographic structure of leucurolysin-A (in red).

The SVMP peptides found in the venoms of *B. atrox* are homologous to venom peptides previously identified in the venom of *B. jararaca* [29]. For instance, the peptide EVWSKKDLIKVEKDSSKTLTSFGEWR (Pep #182, Table 2 and Additional file 1) and its fragments from BATXSVMPII1, BATXSVMPII2 and BATXSVMPII3 (Table 2, Additional file 2) are identical to the corresponding region of the SVMPII insularinase-A [29,43], leucurolysin-A [44], neuwiedase [45], and homologous to several other SVMPs. The peptide EVVYP is the most conserved sequence found, shared among 19 SVMPs of the three classes (Additional file 2). The sequence SFGEWR from the metalloprotease domain is present in 32 of the SVMP peptides (Table 2), suggesting that this region may be exposed to frequent proteolytic processing. The peptides #182 and #32 (ZTLDSFGEWRKTDLLNRKSHDNAQ, Table 2 and Additional file 1) cover a significant homologous region of the SVMPI leucurolysin-A sequence, comprising the amino acids 57 to 96. These peptides were aligned and highlighted in yellow in leucurolysin-A crystallographic structure (4Q1L [44]), as shown in Figure 2A, covering a random coil and an α-helix of the protein not constrained by disulfide bonds. Interestingly, the native 15-aa peptide AHELGHNLGMRHDGN covers the three histidines of the SVMP BATXSVMPII1 catalytic site. This peptide, named Ba1a (Table 2), was also aligned in the 3D structure of leucurolysin-A (in cyan, Figure 2A). The Ba1a sequence contains the consensus motif HEXXHXXGXXH, characteristic of the “metzincin” superfamily of Zn-dependent metalloproteases [46] with three histidines residues (in red) involved in the catalytic Zn-binding region. The Ba1a fold detached from leucurolysin-A structure was simulated and the conformations of the histidines in the peptide differed from the positions in leucurolysin-A (Figure 2B). Although the transcripts of *B. atrox* precursor proteins have been previously reported [47], to our knowledge these native SVMP peptides had not been previously found at the protein level in the venoms. 

**Figure 3.**
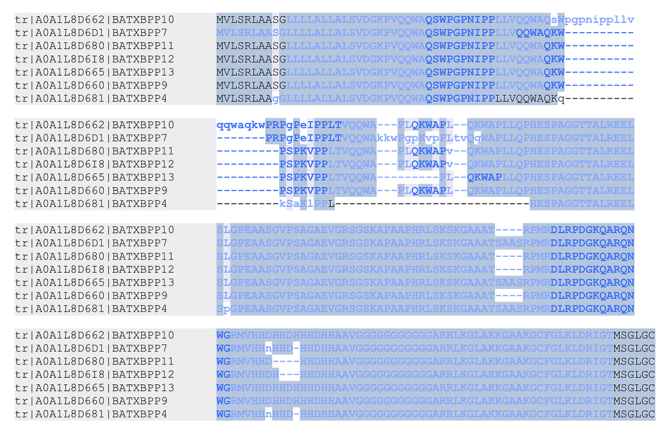
Alignment of the BPP sequences identified in the venoms of *B. atrox* with the precursor proteins. The peptides are shared among seven homologous proteins. Bold blue letters represent the amino acids covered by MS/MS spectra. Capital letters represent complete identity among the aligned sequences. Alignment performed in PEAKS Studio 7.5.

In regard to bradykinin potentiating peptides, *B. atrox* venoms contain the well-known peptide BPP-5a (ZKWAP, in which Z stands for the N-terminal pyroglutamic acid), that provided the basis for the development of important antihypertensive drugs [16,18,48], and its fragment ZKW (Table 2). The peptide ZSWPGPNIPP (BPP-10a) was previously reported in the venom of *B. jararaca* [19] and the ZKWPRPGPEIPP and its fragment ZKWP, in the venoms of *B. atrox* [28] and *B. moojeni* [27]. But we also observed new isoforms of these peptides processed in non-canonical sites, as the sequences ZSWPGPNIP, ZKWPRPGPEIP, ZKWPRPGPEIPPLT and ZQWAQKWPRPGPEIPP. Similar processing was also observed in the venoms of *B. jararaca* [13,29,30,49] and *B. moojeni* [27]. The sequences ZKWPSPKVP and ZKWPSPKVPP are novel BPPs. The ZKWPSPKVPP differs only in the second amino acid from the *B. cotiara*’s ZNWPSPKVPP (BPP-10e) and *B. fonsecai*’s ZRWPSPKVPP (BPP-10f) [29]. The 14 BPPs mapped to 7 protein precursors (Figure 3 and Additional file 2).

**Figure 4.**
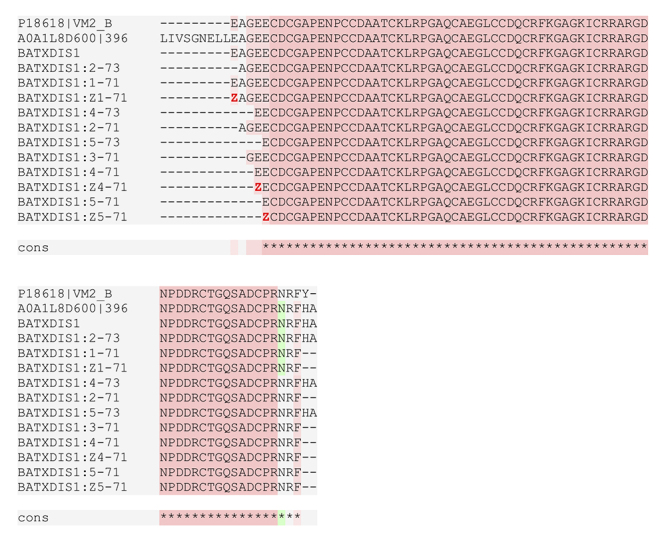
Alignment of 12 disintegrins identified in the venoms of *B. atrox* with BATXPII1 (A0A1L8D600) and batroxostatin (P18618). The sequences were derived from the disintegrin domain of the BATXPII1 precursor. Alignment performed in Tcoffee.

The pooled crude venoms of the two groups (females and males) were split in three separated aliquots and digested with trypsin, Asp-N and Glu-C. The analysis of all digested samples resulted in the identification of additional 1,152 unique cleaved peptides (Additional file 4). The multiple enzyme approach provides a deeper venom proteome coverage. In addition, protein N- and C-terminii consensus can be found by the overlapping of peptides cleaved in different sites and with unexpected amino acids for the enzyme at one of the peptide ends. However, the proteomic analysis was not in the scope of this work and only peptides that assisted in the identification of heavier native peptides were considered. The experimental data of native peptide ions matched the primary structures of 12 proteoforms of the disintegrin derived from BATXPII1 (Figure 4 and Additional file 5). These disintegrins contain the RGD motif and differs from the batroxostatin sequence [21] only in the C-terminal amino acids (FH or FHA instead of FY in the batroxostatin, Figure 4). The disintegrin cotiarin [50] only lacks the C-terminal Ala in comparison to the new BATXDIS1. Consensus analysis of the N- and C-terminal by verification of enzyme cleavages, overlapping peptides, formation of disulfide bonds and comparison of the theoretical monoisotopic masses with the experimental values were used to confirm the identity of these native heavier peptides (Additional file 1and Figure 4). All disintegrins are medium-sized and form 6 disulfide bonds [51].

**Figure 5.**
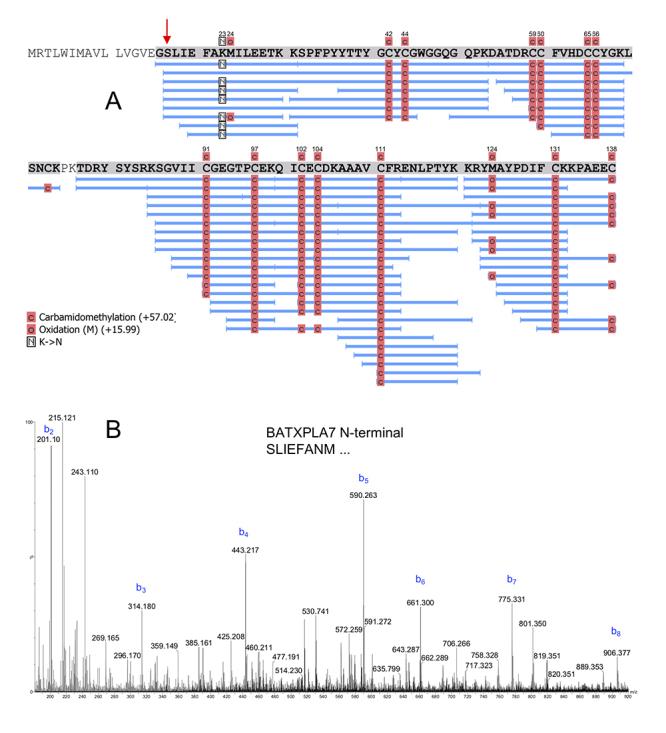
(A) Sequence coverage of the BATXPLA7 showing the consensus of the N-terminal amino acid (S17, indicated by the red arrow) by overlapping of peptides cleaved by different enzymes. The mature sequence presents 123 residues, the K23N mutation and forms seven disulfide bonds. (B) Top-down fragmented MS/MS spectrum of BATXPLA7 and the first eight N-terminal residues determined by *de novo* analysis.

**Figure 6.**
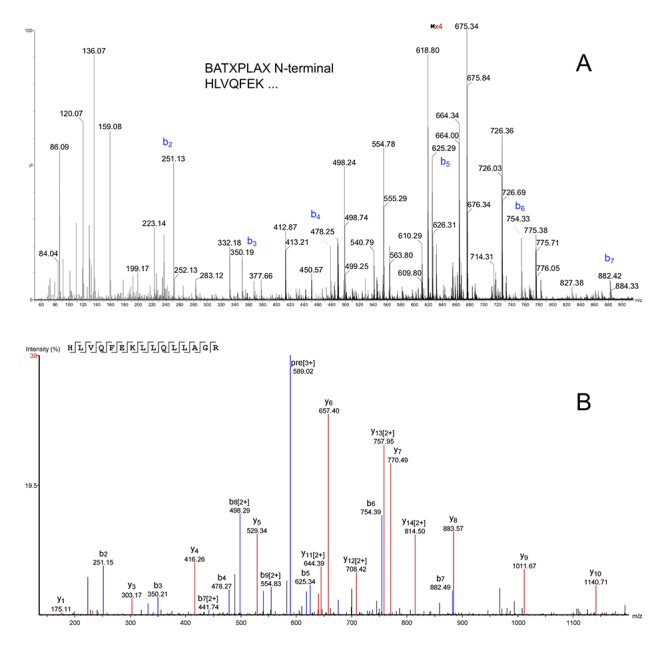
(A) Top-down fragmented MS/MS spectrum of BATXPLA8 and the first seven N-terminal residues determined by *de novo* analysis. (B) Bottom-up MS/MS spectrum and *de novo* analysis of the digested peptide HLVQFEKLLQLLAGR, revealing the 15 N-terminal amino acids of BATXPL8.

This combination of bottom-up and top-down data analysis also revealed the presence of two PLA_2_ sequences, a mutated form of the BATXPLA6, (with the K23N mutation, Figure 5), the D49 PLA_2_ that we denominated BATXPLA7. Top-down fragmentation of multiple charged peaks (Additional file 6) and *de novo* analysis of the MS/MS spectra confirmed the first 8 N-terminal amino acids of the toxin, SLIEFANM (Figure 5). Intact mass analysis shows that it forms 7 disulfide bonds (Additional file 1 and Additional file 6). The presence of BATXPLA6 was not confirmed in the native peptidome data. We identified the peptide GSLIEFANMILEETKK, showing an additional glycine to the BATXPLA7 N-terminus (Additional file 7). However, the peptide ion corresponding to the native extended sequence was not found. As this BATXPLA7 N-terminal peptide was identified by the bottom-up approach, another possibility is that the first Ser was just carbamidomethylated during sample preparation, which results in the same mass difference of a glycine extension (+57.02 Da). The other new PLA_2_, BATXPLA8, also had its first 7 N-terminal amino acids determined by top-down fragmentation of the multiple charged precursor ions (Additional file 6) and *de novo* analysis (Figure 6). Automated d*e novo* analysis of the digested peptide ion at m/z 589.0^+3^ revealed the first 15 N-terminal amino acids of BATXPLA8: HLVQFEKLLQLLAGR (Figure 6). 

In total, 105 native peptides were identified in the venom peptidome of *B. atrox*, in the mass range of 0.4 to 13.9 kDa (Table 2). Thirteen of the heavy sequences (> 7 kDa) were only confirmed after analysis of bottom-up or top-down MS/MS spectra of digested peptides or native peptides, respectively. Although many other proteins were identified in the digested samples of crude venoms, we only considered the peptides that assisted in the assembly of the native peptide structures. It is worth to mention that the *B. atrox* database did not contain glutaminyl-peptide cyclotransferases (GPC), that catalyzes N-terminal pyroglutamate formation. However, by searching the broader *Bothrops* database, we identified the homologous *B. jararaca*’s GPC (Q9YIB5) with 12 peptides and significant score (-10log(p) = 224). 

### ***Bothrops atrox* quantitative peptidomics: females *vs.* males**

**Figure 7.**
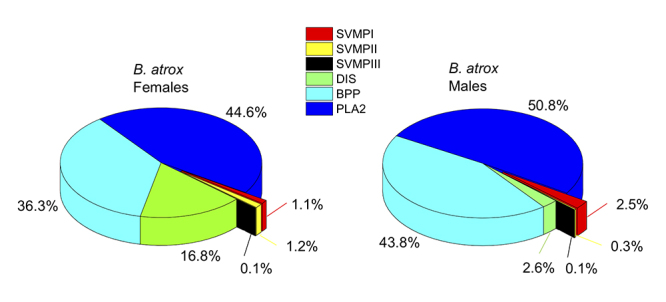
Percentage distribution of peptides by precursor protein families in the venoms of female and male specimens of *B. atrox*. Quantification based on native peptide ion intensities.

**Figure 8.**
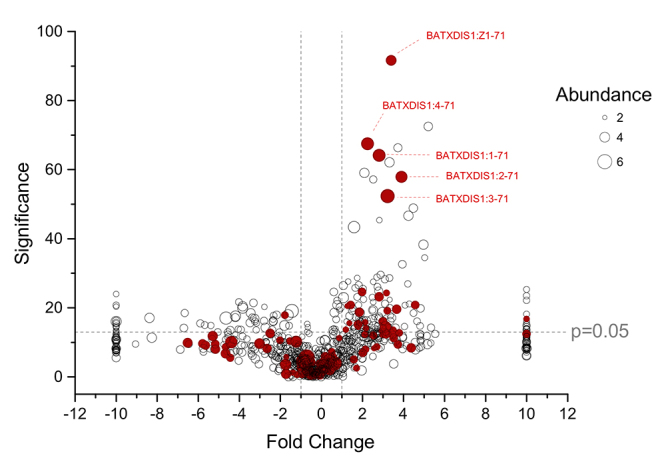
Volcano plot of the peptide ions quantified in the venom of *B. atrox*. Fold changes calculated as the average intensity ratios of female/male and expressed in the log_2_ basis. Abundances in log_10_ scale were proportional to the circle sizes. Filled red circles represent identified peptides and open black circles represent non-identified.

Quantitative analysis of *B. atrox* venom peptides showed that they belong to the following protein families, in decreasing order of abundance: PLA_2_, BPP, DIS and SVMP (Figure 7). Comparison of the profiles in female and male specimens showed a strong difference in the levels of disintegrins, with females presenting 16.8% of these peptides versus 2.6% in males (p < 0.05, Additional file 1). A significant difference was also observed in the levels of SVMPII peptides, 1.2% of peptides in females versus 0.3% in males (p < 0.05, Additional file 1). Although all other peptide families presented statistically equivalent levels (Figure 7), on average, the lack of disintegrins in male venoms are occupied by the BPPs, showing 43.8% of the peptides, against 36.3% in females. Individually, from the 105 identified peptides, 26 peptides are differentially expressed at significant levels and 25 of these 26 are increased in females (Figure 8). 

The overall quantitative profile of the disintegrin family is a reflection of the individual peptides. All seven significantly different DIS peptides are highly increased in the venoms of females (Table 2 and Figure 8). For instance, the peptides BATXDIS1:1-71 and BATXDIS1:3-71, on average the 3^rd^ and 5^th^ most intense ions of the peptidome, are 7 and 9 times more intense in the venoms of females, respectively. BATXDIS1:Z1-71, BATXDIS1:4-71 and BATXDIS1:2-71 are also among the most significant differential peptides. Nine of the 18 differential SVMP peptides contain the sequence SFGEWR, all increased in females. The only identified peptide increased in males is the BATXSVMPI1 fragment VEIWSNKDLINVQPAAP. There are other peptide ions increased in males, however these were not identified (Figure 8). 

## Discussion

Venoms of *Bothrops* snakes are rich in biologically active peptides that play important roles in the envenomation process. BPPs, for instance, target the cardiovascular system of the prey by inhibiting the angiotensin-converting enzyme (ACE) [52]. ACE participates of blood pressure regulation by cleaving angiotensin I to angiotensin II [53], an hypertensive peptide, and by inactivating the hypotensive peptide bradykinin (Bk) [54]. The synergistic action of endogenous Bk generation by venom proteases and inhibition of ACE by the BPPs may cause a vascular shock in mammal preys [55]. Although a higher percentage of BPPs was observed in males of *B. atrox* (44% vs. 36%), the difference was not statistically significant (Figure 7). BPPs seem to be equally important to both genders of *B. atrox*, presenting high percentages of the peptidomes. One characteristic of the BPPs is the pyroglutamate at the N-terminal [17,31], whose formation is catalyzed by GPC. The identification of the GPC (Q9YIB5) in the venom of *B. atrox* explains the high number of BPPs identified.

Snake venom disintegrins containing the RGD motif are potent inhibitors of aggregation responses due to the binding to platelet α_IIb_β_3_ integrins [21,56]. In the *Bothrops* genus, these toxins have been reported in the venoms of several species as *B. atrox* [21], *B. cotiara* [50,57], *B. jararaca*, *B. jararacussu* [50], *B. asper* [58], *B. insularis* [43] and *B. colombiensis* [59], for instance. These type of disintegrins are proteolytically processed from PII SVMPs and released as stable proteins [60]. We observed significantly higher levels of disintegrins in the *B. atrox* venoms of females, 6.5 times higher than males (Figure 7). Such higher level of disintegrins in females should reflect on higher inhibition of platelet aggregation on preys, and consequently to higher anticoagulant activity. However, minimum coagulant dose assays with crude venoms of *B. atrox* showed just the opposite effect, as the venoms of females presented high coagulant activity in citrated human plasma [14]. To interpret the result, it is important to consider that proteins as SVSP, SVMP, CTL, PLA_2_ and other toxins play roles in the coagulation process. Some are procoagulant and others are anticoagulant [61]. Thus, synergistic effect [14,62] and a balance of actions produce the final venom activity. Possibly, specific platelet aggregation assays could be used to evaluate the activities of disintegrins of the different genders.

Several other SVMP peptide fragments are observed in *B. atrox* venoms. They represent smaller percentages of the peptidomes, but may play relevant biological roles. We identified the 15-aa peptide Ba1a containing the three histidines of the SVMP BATXSVMPII1 catalytic site. Computational fold simulation indicates slight positional shifts of the first two Ba1a histidines from His-142 and His-146 of the template SVMP leucurolysin-A (Figure 2B). However, the third His of Ba1a turns considerably in comparison to the corresponding His-152 of leucurolysin-A. This latter shift may affect the Zn affinity of the peptide and modify its biological action in comparison to the original protein. The biological activity of Ba1a can be explored in future experimental studies. Anyway, it is interesting to observe the SVMP catalytic site in this native peptide. The SVMP peptides may have been originated from proteolytic processing of proteases inside the venom glands, as the SVMPs and SVSPs [29,31]. The hypothesis of peptides being produced endogenously is corroborated by the identification of the GPC (Q9YIB5) in the crude venom and the relatively high frequency of pyroglutamic acid in the N-terminal of the SVMP peptides (Table 2). Unexpectedly, L-amino acid oxidase peptides were not found in the venoms of *B. atrox* as opposed to other studies of *Bothrops* snake venom peptidomes [29,30,63]. The use of protease inhibitors immediately after venom extraction may have prevented the generation of artefactual peptides, as previously reported [29]. 

Although this was a peptidomic study, we also observed 13.8 kDa PLA_2_ toxins in our analyses. There is not an official definition of a peptide size, although most studies use the 10 kDa as an approximate cut-off value [64]. Nevertheless, the *B. atrox* PLA_2_ toxins were sufficiently hydrophilic to be extracted in our peptide enrichment methods. PLA_2_ represented the most abundant peptide family of both genders with 45% and 51% of the total venom peptides in females and males, respectively. The higher percentage of PLA_2_ in males was also observed in the proteomic study and in the *in vitro* activity by colorimetric assay [14]. It is important to mention that the peptidomic quantification was based in native precursor ion intensities. While the quantitative methods were different, the corroboration of proteomic, peptidomic and biological activity data for PLA_2_ is noteworthy.

## Conclusion

The venom peptidomes of male and female specimens of *Bothrops atrox* were uncovered by mass spectrometry-based approaches in the present work. New peptides were identified as well as known peptides processed at non-canonical sites were observed. The genders present abundant and statistically equivalent levels of BPPs and PLA_2_, but female venoms are significantly richer in disintegrins. This difference may result in biological implications on platelet function in preys; however, current experimental data do not point to differences in coagulation. Specific assays should be performed in future works to elucidate possible differences on platelet aggregation by male and female venoms. It was also shown that SVMP peptides are probably processed endogenously due to the presence of pyroglutamic fragments and of GPC in the venom. In summary, the differences in the venom peptidomes may reflect on distinct ecological needs of males and females and may have their potential pharmacological properties explored in future works. 

## Supplementary material

The following online material is available for this article:

Additional file 1. Peptide ions obtained by LC-MS/MS runs of *Bothrops atrox* venom peptidomic samples. A total of 16 runs were performed on the venoms of males and female specimens.

Additional file 2. Significant peptides identified in the venoms of *Bothrops atrox* and the originating proteins. Homologous peptides are shared by several proteins.

Additional file 3. Venn diagram of unique peptides identified by database search of the *B. atrox* peptidome LC-MS/MS data. Green: *B. atrox* database; blue: *Bothrops* database; pink: Serpentes database. Venn diagram plot in Jvenn.

Additional file 4. Peptides identified in the crude venoms of *Bothrops atrox* digested with the enzymes Asp-N, Clu-C and trypsin. Database search performed in PEAKS Studio 7.5 against *B. atrox* venom proteins. Peptide scores are presented in the columns.

Additional file 5. Isotopic patterns of six of the most intense *B. atrox* disintegrins with multiple charges.

Additional file 6. ESI-MS spectra of multiple charged peaks of *B. atrox* PLA_2_ toxins showing average m/z values. **(A)** BATXPLA7; **(B)** BATXPLA8.

Additional file 7. ESI-MS/MS spectrum of the *B. atrox* PLA_2_ peptide SLIEFANMILEETKK. The N-terminal GS may also be the carbamidomethylated S, as both have the same mass. Peptide ion observed at m/z 608.33^+3^.
