# Supplementary material for: Comparative gender peptidomics of Bothrops atrox venoms: are there differences between them?
Source: J Venom Anim Toxins Incl Trop Dis. 2020 Oct 7;26:e20200055. doi: 10.1590/1678-9199-JVATITD-2020-0055 (PMC7546584; doi:10.1590/1678-9199-JVATITD-2020-0055)

J Venom Anim Toxins incl Trop Dis
https://doi.org/10.1590/1678-9199-JVATITD-2020-0055

Supplementary Material to "Comparative gender peptidomics of *Bothrops atrox* venoms: are there differences between them?"

Additional file 3. Venn diagram of unique peptides identified by database search of the *B. atrox* peptidome LC-MS/MS data. Green: *B. atrox* database; blue: *Bothrops* database; pink: Serpentes database. Venn diagram plot in Jvenn.

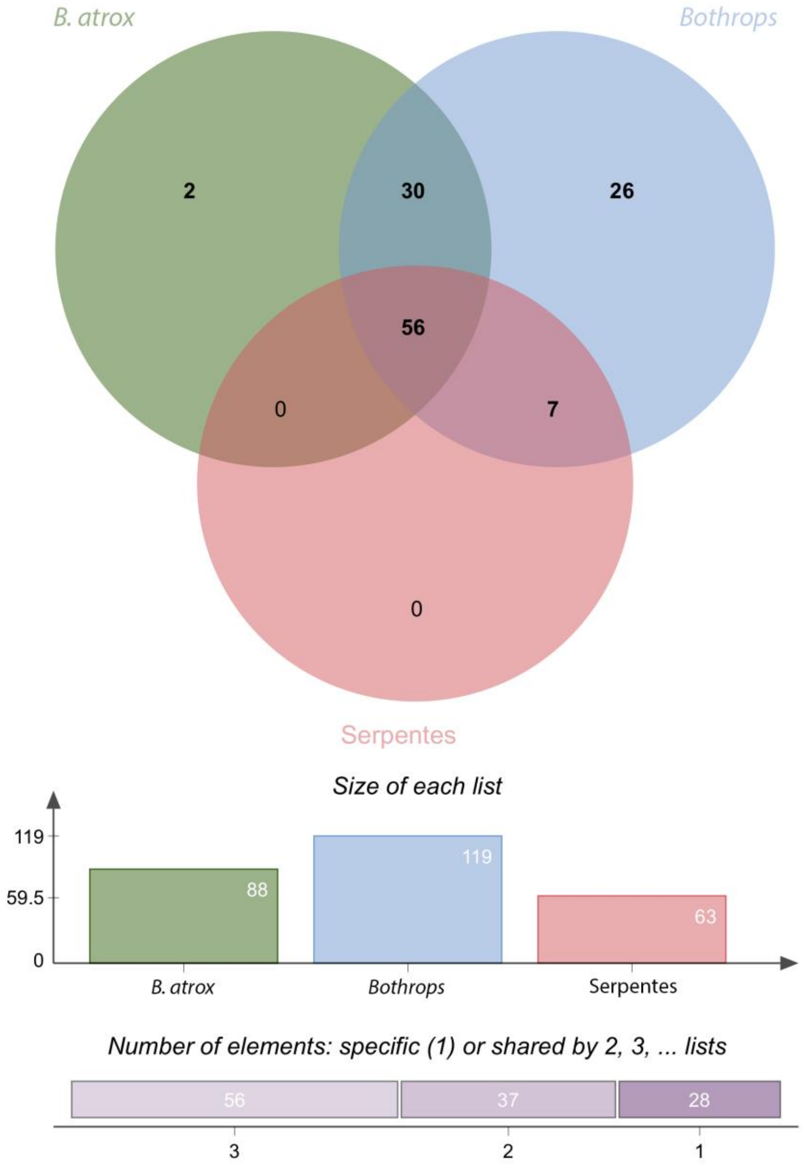

Supplement: Additional file 3. [file 1678-9199-jvatitd-26-e20200055-s3.pdf]
